# The efficacy of virtual reality in adults during puncture biopsy: A protocol of a systematic review and meta-analysis

**DOI:** 10.1371/journal.pone.0316260

**Published:** 2024-12-30

**Authors:** Xueling Qiu, Lukun Sun, Lu Tang, Xiaochen Jiang, Weifeng Wang, Fan Sun, Chenxi Sun, Tianyi Song

**Affiliations:** 1 Department of Stomatology, The 960th Hospital of People’s Liberation Army of China (PLA), Jinan, China; 2 School of Nursing, Shandong First Medical University (Shandong Academy Of Medical Sciences), Taian, China; 3 Department of Rehabilitation, The 960th Hospital of People’s Liberation Army of China (PLA), Jinan, China; 4 School of Nursing, Shandong Second Medical University, Weifang, China; 5 School of Nursing, Shandong University of Traditional Chinese Medicine (TCM), Jinan, China; 6 School of Nursing, Jinzhou Medical University, Jinzhou, China; 7 Department of Medical Engineering, The 960th Hospital of People’s Liberation Army of China (PLA), Jinan, China; University of Perugia: Universita degli Studi di Perugia, ITALY

## Abstract

**Background:**

Puncture biopsy is a primary method for obtaining tissue or cell samples from tumors for histopathological diagnosis. However, patients often experience pain, anxiety, and discomfort during the procedure. Virtual reality is a novel technology developed through advancements in computer skill, and it is utilized in healthcare as a cognitive approach to relieve pain and relaxation. However, there is controversy in published trials regarding the efficacy of virtual reality in adults during puncture biopsy. Therefore, a synthesis of objective and accurate data is needed to support the efficacy of virtual reality for pain relief during puncture biopsy in adults.

**Method and analysis:**

To identify suitable randomized controlled trials, published literature in eight electronic databases will be searched, including PubMed, Web of Science, Cochrane Library, Scopus, EMBASE, Chinese National Knowledge Infrastructure (CNKI), Wan-fang Data, and Chinese Biomedical Database (CBM). The collected data will be consolidated and subjected to meta-analysis by using RevMan 5.4. Mean difference will describe the continuous variables. 95% confidence intervals (CI) will characterize the interval estimates. Model categories will be selected based on heterogeneity. The quality of the inclusion of randomized controlled trials in terms of methodological quality will be assessed by the Cochrane Risk of Bias Assessment Tool. Additionally, strength and certainty of the evidence will be assessed by the GRADE system.

**Discussion:**

The following protocol delineates the fundamental process and methodology to be employed in a systematic review and meta-analysis of data pertaining to the efficacy of virtual reality in the context of adult puncture biopsy. The results of the study will furnish healthcare professionals with evidence-based clinical evidence, thereby facilitating sound clinical decision-making and yielding beneficial consequences for the clinical domain.

**Trial registration:**

**PROSPERO registration number:** CRD42024539303.

## Introduction

Puncture biopsy represents a principal methodology for the acquisition of tissue or cellular samples from neoplastic lesions, facilitating the histopathological diagnosis of such lesions. It is the preferred standard of care for the initial assessment of cells from neoplastic lesions and other lesions, providing diagnostic insights [1, 2]. However, because it is an invasive procedure, a large proportion of patients who underwent puncture biopsy experienced significant morbidity associated with the surgery, including pain, anxiety, and other forms of discomfort [3]. The experience of pain and anxiety during puncture biopsy represents a significant burden for many patients. Anxiety often coexists with pain, and exacerbates the perception of pain [4]. In initial studies conducted on prostate biopsy without anesthesia, it was observed that between 65 to 90% of patients reported experiencing discomfort, with 30% of these individuals reporting significant pain [5, 6]. Similarly, numerous studies have indicated that between 70 and 93.4% of patients who have undergone bone marrow aspiration and biopsy procedures have experienced pain. Furthermore, approximately 36 to 59.2% of these patients have reported that the pain was moderate to severe [7–10]. Thus, pain is one of the most significant challenges encountered during procedure and needs to be addressed urgently. The effective management of pain during the procedure is of critical importance in order to ensure patient compliance. Furthermore, it may lead to an improvement in patient satisfaction and acceptability of re-biopsy.

Currently, local anesthesia is used as a common analgesic modality for the management of pain in patients undergoing puncture biopsy [11]. However, some studies have demonstrated that local anesthesia is not an effective method of providing adequate analgesia [12, 13]. Besides, opioids or benzodiazepines are also applied to relieve pain and anxiety during puncture biopsy [8, 11]. However, these analgesic and sedative drugs do not relieve all pain during procedure or the anxiety associated with subsequent recall of pain, and can bring about side effects such as low blood pressure, bradycardia, nausea, vomiting, and prolonged hospitalization time [14, 15]. Inadequate analgesia and side effects of drugs may affect the compliance of patients with procedures, as well as subsequent diagnosis and treatment of the diseases, and hinder patients compliance with a future biopsy.

With the rise of non-pharmacologic treatments, various non-pharmacologic approaches are gradually being used to puncture biopsy. A meta-analysis revealed that music therapy was associated with a reduction in pain and anxiety during breast biopsies [16]. Similarly, in a randomized controlled study conducted by Hızlı et al., it was found that a ephemeral preoperative hypnosis intervention can significantly alleviate anxiety and pain in patients during prostate puncture biopsy [17]. However, the implementation of music therapy and hypnosis may necessitate the acquisition of comprehensive training by interventionists to facilitate the creation of bespoke experiences, a requirement that may be constrained by the limitations of many medical settings [18]. Meanwhile, in the current era of rapidly advancing technology, these traditional non-pharmacological interventions may prove inadequate in meeting the needs and expectations of patients.

In light of the significant prevalence and intensity of pain associated with puncture biopsies, and the constraints of conventional analgesic approaches, the advent of novel technologies, such as virtual reality, presents a promising avenue for the management of pain and anxiety. Virtual reality (VR) is a novel technology that has been developed as a result of advances in computer technology, and it is being employed in the field of medical as a cognitive approach to pain relief and relaxation [19]. The use of VR for analgesia was first described during acute burns dressing changes in 2000, and the preliminary results of the study found that immersive VR may represent a potentially viable treatment for acute pain that deserved more attention [20]. In regard to clinical applications, a number of studies have indicated that VR can be employed as a supplementary or alternative non-pharmacological analgesic for a variety of pain-inducing procedures and in the alleviation of chronic pain [21]. Theoretically, when a healthcare professional performs an invasive procedure, the patients will no longer feel stuck in the real world of pain, but in another pleasurable, three-dimensional virtual world [22].

Despite several early randomized controlled trials that attempted to evaluate data related to the effectiveness of VR for analgesia during puncture biopsy, the results of the studies remain controversial. Furthermore, the conclusions of the studies lack rigorous, conclusive data to support the efficacy of VR for analgesia during puncture biopsy. Consequently, the evidence is insufficient to recommend VR as one of the effective therapies for pain management in adults during puncture biopsy. Accordingly, the objective of this study was to conduct a comprehensive systematic review of the existing literature to investigate the analgesic efficacy of VR in puncture biopsy procedures in adults. Furthermore, it may complement the evidence for the effectiveness of VR in pain management, with broad implications for the clinical use of VR.

### Research question

Does virtual reality technology provide pain and anxiety relief for adults during puncture biopsy?

## Methods and analysis

### Study registration

In order to enhance the transparency and rigor of this study, the Preferred Reporting Items for Systematic Reviews and Meta-Analyses Protocols (PRISMA-P) guideline is rigorously adhered to [23] (S1 Table). The study has been registered on the Prospero (CRD42024539303).

Eligibility criteria

The PICOS framework will be used in our study:

#### Participants

The study will include patients aged 18 years and above who receive VR during puncture biopsy. No restrictions will be placed on the gender, country, or race of the participants.

#### Intervention

The primary intervention is VR in the intervention group. There are no restrictions on the type of VR experience, the content to be viewed, or the duration of the viewing period.

#### Comparator

The primary intervention is other treatments without VR in the control group.

1. Analgesic drugs: local anesthetics, N_2_O, etc.
2. Other methods of distraction.
3. No intervention: routine treatment, routine care.

#### Outcomes

According to the above criteria, qualified studies must document at least one of the following results:

#### Primary outcome

The primary outcome will be the level of pain experienced by the subject after using VR or alternative treatments. The pain score will be expressed using visual analogue scale (VAS), numerical rating scale (NRS), or other alternative scores.

#### Secondary outcome

The secondary outcome is the level of anxiety experienced by the subject following the use of VR or alternative treatments. Anxiety level is quantified using the State-Trait Anxiety Inventory (STAI). The STAI comprises two distinct scales, each employing a 4-point Likert format, for the assessment of state and trait anxiety. The rating scale ranges from 1 (no anxiety) to 4 (fully anxious), with each scale comprising 20 items [24]. The total score obtained from the scale ranges from 20 to 80, with higher scores indicating elevated anxiety levels. The State Anxiety Inventory (STAI-S) requests that respondents describe their feelings at a specific point in time and in a specific context. In contrast, the Trait Anxiety Inventory (STAI-T) requires that respondents indicate their general feelings about anxiety [25]. The initial 20 items assess state anxiety, while the subsequent 20 items evaluate trait anxiety [26].

#### Study design

The study will include only randomized controlled trials (RCTs) that investigated the efficacy of VR in adults during puncture biopsy. Studies that do not adhere to the RCT design, including case series, reviews, and other non-RCT study types, will not be included in this study.

### Information sources

From the creation of databases through October 2024, the published literature in eight electronic databases will be searched, including PubMed, Web of Science, Cochrane Library, Scopus, EMBASE, Chinese National Knowledge Infrastructure (CNKI), Wan-fang Data, and Chinese Biomedical Database (CBM). In addition, the systematic review and meta-analyse pertaining to VR will be subjected to rigorous scrutiny. Moreover, the references of each study will be examined to identify any studies that may have been inadvertently omitted.

### Search strategy

The review process will be performed by two independent reviewers (XLQ and WFW), with any discrepancies to be settled through consultation through another reviewer (XCJ). The retrieval approach will encompass three fundamental elements: intervention (e.g., virtual reality or virtual reality exposure therapy or VR); participants (e.g., puncture biopsy or biopsy or puncture). In order to guarantee an efficacious search, a combination of medical subject headings (MeSH) and free words will be employed, and bespoke retrieval patterns will be implemented for each database. The search strategy is exemplified by PubMed, as illustrated in Table 1. Considering the different requirements of different databases, the search strategy can be adjusted as needed. For further details regarding the other search strategies, please refer to S2 Table. Moreover, In order to ensure that this review is kept up to date with the latest developments, we will conduct further research on the World Health Organization’s International Clinical Trials Registry Platform (https://www.who.int/clinical-trials-registry-platform), the Chinese Clinical Trial Registry (http://www.chictr.org.cn), and the Clinical Trials Registry (https://clinicaltrials.gov/). This will help us to identify any ongoing eligible RCTs that may have been overlooked.

**Table 1:** The search strategy for PubMed.

ORDER	STRATEGY
#1	Virtual reality [MeSH Terms]
#2	Virtual reality exposure therapy[MeSH Terms]
#3	(Virtual reality immersion therapy OR Virtual reality therapy OR VR)[Title/Abstract]
#4	#1 OR #2 OR #3
#5	Biopsy, Needle[MeSH Terms]
#6	(Puncture Biopsy OR Puncture OR Biopsy)[Title/Abstract]
#7	#5 OR #6
#8	#4 AND #7

### Study selection process

**Fig 1:**
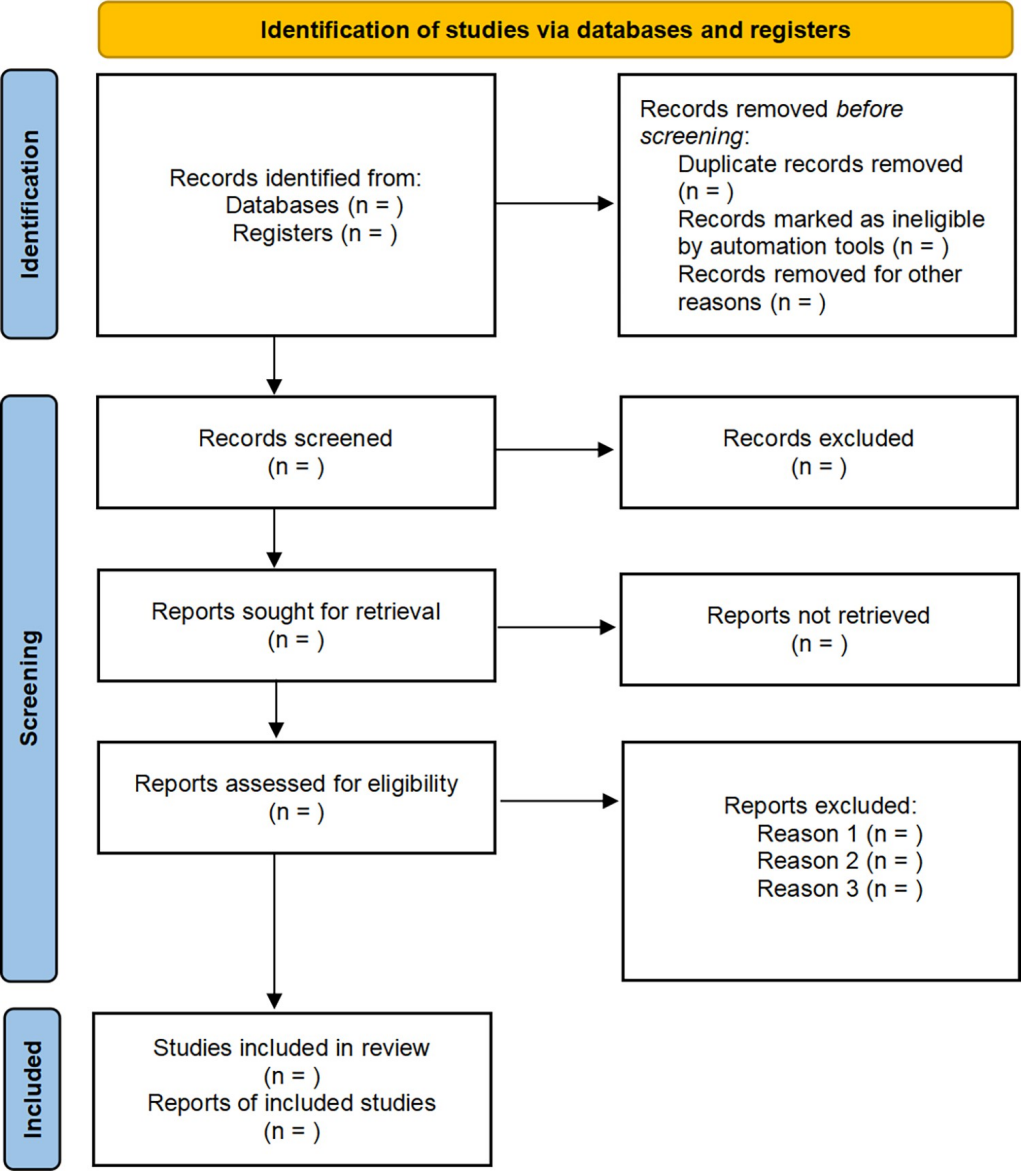
The PRISMA flow diagram of the study selection process.

Two reviewers (XLQ and WFW) will conduct the literature search independently. The search data will be imported to Endnote X9, where they will be subjected to a deduplication process. The study screening process will be performed in two stages. Initially, studies that may fulfill the established inclusion criteria will be selected based on an evaluation of the title and abstract sections of the literature. Subsequently, two reviewers (XLQ and WFW) will identify trials that meet the inclusion criteria based on a thorough examination of the studies’ full texts. In the event of a discrepancy between the two reviewers (XLQ and WFW) regarding the selection of studies, the third reviewer (XCJ) will be consulted to arbitrate the dispute. The specific screening process is shown in Fig 1.

### Data extraction and data items

The data extracted from the source material includes author name, publication years, country, study design, participants characteristics, operation type, sample sizes, adjuvant analgesics and any outcomes, including primary and secondary outcomes. If the original publication lacks pertinent data, the reviewer will contact the author via electronic mail to request the missing information. And if the authors are unable to be reached, these studies will be excluded from our analysis. A limited analysis will be conducted using accessible data, and the potential implications of any missing information will be considered.

### Methodological quality assessment

Two independent reviewers (XLQ and WFW) will assess the risk of bias for all included studies by using the Cochrane Risk of Bias Assessment Tool [27]. The quality of each study will be assessed against seven main criteria. The level of risk will be classified into three categories: ‘low’, ‘high’ and ‘unclear’. Should the committee be unable to reach a consensus, the third researcher (XCJ) will be consulted.

### Data synthesis and statistical analysis

This review will use the RevMan 5.4 software (Review Manager version 5.4) for the analysis of the data. Continuous variables will be expressed as mean differences (MD). In instances where disparate measurement instruments are employed to assess a given variable, the standardized mean difference (SMD) will be utilized for analytical purposes. Interval estimates will be expressed as 95% confidence intervals (CI). The degree of statistical heterogeneity between trials will be evaluated using the *I*^*2*^ statistic. If the results have significant heterogeneity, we will investigate the underlying causes through subgroup analysis. Furthermore, we will assess the stability and reliability of the results through sensitivity analysis. If the heterogeneity persists at a high level, random effects models will be selected. In the event that a quantitative meta-analysis is not deemed appropriate for this study, the results will be presented and analyzed in qualitative form only.

#### Trial sequential analysis

In order to mitigate the potential for randomization error, we will utilize the TSA 0.9.5.10 Beta version. TSA also estimates the required information size (RIS) for a meta-analysis to yield stable conclusions. This provides a termination criterion for clinical trials to reach a specified sample size. The results will be monitored by RIS, cumulative Z-curve, and TSA monitoring boundaries. The bilateral type I error rate will be maintained at 5% (α boundaries), the β will be set at 20% (80% power), and a clinically significant difference of 20% will be assumed.

#### Subgroup analysis

Once the requisite number of samples and data have been collected for the relevant subgroups, exploratory subgroup analyses will be conducted to explore potential causes of heterogeneity. These analyses will be conducted across subgroups based on the following criteria:

1. The type of procedures.
2. Gender (male, female).
3. The type of control groups.
4. Local anesthesia.
5. Modes of VR.

#### Sensitivity analysis

When sufficient studies are available, sensitivity analysis is needed for studies. Studies with quality flaws and large sample sizes will be excluded to check the stability of the final results.

### Assessment of the quality of evidence

Two reviewers (XLQ and WFW) will evaluate the quality of the evidence pertaining to all outcomes. We will assess the quality of evident in accordance with the Cochrane Grading of Recommendations, Appraisals, Developments and Evaluations (GRADE) methodology [28]. The strength of the evidence will be evaluated on a four-point scale: high: moderate, low, and very low. The quality of evidence is a measure of the degree of confidence that can be placed in the accuracy of an estimated effect. In conclusion, the results of the study will be presented in tabular form in the final publication.

### Publication bias

If the study encompasses a minimum of 10 randomized controlled trials for meta-analysis, the Egger’s test and funnel plot will be employed to assess the potential for publication bias. In the event that publication bias is suspected, the Duvall and Tweedle trim-and-fill model will be employed for the purpose of adjusting effect estimates.

### Ethical consideration

The project does not need ethical approval, as the results of the study will be published in a peer-reviewed publications, and participants’ personal data will be excluded from systematic reviews and meta-analyses.

## Discussion

Puncture biopsy represents a significant methodology for the identification and analysis of pathological tissue and cellular patterns, thereby facilitating the formulation of a pathological diagnosis. And it is important for the early diagnosis and treatment of clinical diseases [29].

Nowadays, most biopsies are performed under continuous local anesthesia, such as liver biopsy. Nevertheless, a considerable number of patients have reported that local anaesthetics are insufficient for providing adequate pain relief during puncture biopsies. Inadequate analgesia may result in patients refusal to undergo a re-biopsy without analgesia, leading to delay in the early diagnosis and treatment of the patients’ diseases. With the increased emphasis on pain management during puncture biopsy, the widespread use of non-pharmacologic interventions offers new ideas and new prospects for pain management during puncture biopsy. In recent times, VR has been identified as a promising new avenue for the management of discomfort and anxiety in a variety of procedures within the medical field [30]. The application of visual or auditory distraction techniques has been demonstrated to have a beneficial impact on the reduction of pain, the lowering of heart rate, blood pressure, body temperature, and respiratory rate, the facilitation of relaxation in patients, and the enhancement of quality of life. Moreover, the viewing of videos featuring natural settings and sounds has been demonstrated to induce relaxation by triggering the release of serotonin, enhancing feelings of well-being, and reducing blood pressure [31]. Currently, some clinical trials have demonstrated the positive effects of VR in relieving pain and anxiety during puncture biopsy. Korkmaz et al. discovered that viewing VRG video streaming can diminish the discomfort and distress experienced by patients during bone marrow aspiration and biopsy procedures. Their findings revealed statistically significant discrepancies between the experimental and control groups [26]. Similarly, VR has the same effectiveness and usefulness as prostate puncture biopsy [32]. Nevertheless, Prabhu et al. discovered that anxiety during breast puncture biopsy is markedly diminished in the VR intervention group relative to the standard-of-care control group. However, when post-intervention pain scores are compared, no significant discrepancy is observed between the two groups [33]. Therefore, the current clinical findings are not sufficient to support the analgesic effectiveness of VR application in adults during puncture biopsy. Consequently, the objective of this study is to demonstrate the analgesic efficacy of VR in reducing pain associated with puncture biopsy procedures in adults. The findings of this study will help to get a conclusion about the efficiency of VR for pain management during puncture biopsy.

When future results of this study are confirmed, we will further enrich effective treatments for pain relief during puncture biopsy and further clarify the effectiveness of VR in providing clinical decisions based on evidence-based medicine. However, there may be some problems with the operationalization of this study. Firstly, because the inclusion of the population is limited to adults, it may not be possible to clarify the effectiveness in other populations. Secondly, because we did not limit the general characteristics of VR technology at the time of inclusion in the study, its intrinsic complexity and variability, including factors such as differences in viewing content and headset quality, may affect the reliability of the results. In addition, heterogeneity may also be a result of this phenomenon.

## Conclusions

In conclusion, this protocol delineates the fundamental process and anticipated methodology for a systematic review and meta-analysis of data on the efficacy of VR in puncture biopsy procedures in adults. The results of the study will furnish evidence-based clinical evidence to assist healthcare professionals in making clinical decisions, with positive implications for clinical practice.

## Supporting information

S1 Table PRISMA-P (Preferred Reporting Items for Systematic review and Meta-Analysis Protocols) 2015 checklist: Recommended items to address in a systematic review protocol. (DOCX)

S2 Table Search strategies. (DOCX)
