# Supplementary material for: The efficacy of virtual reality in adults during puncture biopsy: A protocol of a systematic review and meta-analysis
Source: PLoS One. 2024 Dec 30;19(12):e0316260. doi: 10.1371/journal.pone.0316260 (PMC11684728; doi:10.1371/journal.pone.0316260)
Supplement: S2 Table — (DOCX) [file pone.0316260.s002.docx]

**S2** **Search** **strategies**

| Database | Search number | Search terms |
| --- | --- | --- |
| Pubmed | #1 | Virtual reality [MeSH Terms] |
|  | #2 | Virtual reality exposure therapy[MeSH Terms] |
|  | #3 | (Virtual reality immersion therapy OR Virtual reality therapy OR VR)[Title/Abstract] |
|  | #4 | #1 OR #2 OR #3 |
|  | #5 | Biopsy, Needle[MeSH Terms] |
|  | #6 | (Puncture Biopsy OR Puncture OR Biopsy)[Title/Abstract] |
|  | #7 | #5 OR #6 |
|  | #8 | #4 AND #7 |
| Web of Science | #1 | TS=(virtual reality) OR TS=(VR) OR TS=(Virtual reality exposure therapy) OR TS=(Virtual reality immersion therapy) OR TS=(Virtual reality therapy) |
|  | #2 | (((TS=(Biopsy, Needle)) OR TS=(Puncture Biopsy)) OR TS=(Puncture)) OR TS=(Biopsy ) |
|  | #3 | #1 AND #2 |
| EMBASE | #1 | 'virtual reality'/exp |
|  | #2 | 'virtual reality exposure therapy'/exp |
|  | #3 | #1 OR #2 |
|  | #4 | 'needle biopsy'/exp |
|  | #5 | 'puncture'/exp |
|  | #6 | 'biopsy'/exp |
|  | #7 | #4 OR #5 OR #6 |
|  | #8 | #3 AND #7 |
| SCOPUS | #1 | (TITLE-ABS-KEY("virtual reality") OR TITLE-ABS-KEY("virtual reality exposure therapy") OR TITLE-ABS-KEY("virtual reality immersion therapy") OR TITLE-ABS-KEY ("virtual reality therapy") OR TITLE-ABS-KEY(VR)) |
|  | #2 | ( TITLE-ABS-KEY ( "biopsy, needle" ) OR  TITLE-ABS-KEY ( "puncture biopsy" ) OR TITLE-ABS-KEY ( puncture ) OR TITLE-ABS-KEY ( biopsy ) ) |
|  | #3 | #1 AND #2 |
| Cochrane Library | #1 | (virtual reality) OR (VR) OR (virtual reality exposure therapy) OR (virtual reality immersion therapy) OR (virtual reality therapy) (Word variations have been searched) |
|  | #2 | (puncture biopsy) OR (puncture) OR (biopsy) OR (biopsy, needle) (Word variations have been searched) |
|  | #3 | #1 AND #2 |
| Chinese National Knowledge Infrastructure (CNKI) | #1 | 主题：虚拟现实 + 虚拟现实技术 + VR |
|  | #2 | 主题：穿刺 + 活检 + 穿刺活检 + 穿刺活检术 + 针穿刺 |
|  | #3 | #1 AND #2 |
| Wan-fang Data | #1 | 主题：虚拟现实 OR 虚拟现实技术 OR VR |
|  | #2 | 主题：穿刺 OR 活检 OR 穿刺活检 OR 穿刺活检术 OR 针穿刺 |
|  | #3 | #1 AND #2 |
| Chinese Biomedical Database (CBM) | #1 | ( "虚拟现实"[标题:智能] OR "虚拟现实技术"[标题:智能] OR "VR"[标题:智能]) AND( "穿刺"[标题:智能] OR "活检"[标题:智能] OR "穿刺活检"[标题:智能] OR "穿刺活检术"[标题:智能] OR "针穿刺"[标题:智能]) |
